# AI-Aided Gait Analysis with a Wearable Device Featuring a Hydrogel Sensor

**DOI:** 10.3390/s24227370

**Published:** 2024-11-19

**Authors:** Saima Hasan, Brent G. D’auria, M. A. Parvez Mahmud, Scott D. Adams, John M. Long, Lingxue Kong, Abbas Z. Kouzani

**Affiliations:** 1 School of Engineering, Deakin University, Geelong, VIC 3216, Australia; hasansai@deakin.edu.au (S.H.); bdauria@deakin.edu.au (B.G.D.); scott.adams@deakin.edu.au (S.D.A.); john.long@deakin.edu.au (J.M.L.); 2 Faculty of Science, University of Technology Sydney, Ultimo, NSW 2007, Australia; parvez.mahmud@uts.edu.au; 3 Institute for Frontier Materials, Deakin University, Geelong, VIC 3216, Australia; lingxue.kong@deakin.edu.au

**Keywords:** wearable device, conductive hydrogel, strain sensor, triboelectric nanogenerator, 1D CNN, gait analysis

## Abstract

Wearable devices have revolutionized real-time health monitoring, yet challenges persist in enhancing their flexibility, weight, and accuracy. This paper presents the development of a wearable device employing a conductive polyacrylamide–lithium chloride–MXene (PLM) hydrogel sensor, an electronic circuit, and artificial intelligence (AI) for gait monitoring. The PLM sensor includes tribo-negative polydimethylsiloxane (PDMS) and tribo-positive polyurethane (PU) layers, exhibiting extraordinary stretchability (317% strain) and durability (1000 cycles) while consistently delivering stable electrical signals. The wearable device weighs just 23 g and is strategically affixed to a knee brace, harnessing mechanical energy generated during knee motion which is converted into electrical signals. These signals are digitized and then analyzed using a one-dimensional (1D) convolutional neural network (CNN), achieving an impressive accuracy of 100% for the classification of four distinct gait patterns: standing, walking, jogging, and running. The wearable device demonstrates the potential for lightweight and energy-efficient sensing combined with AI analysis for advanced biomechanical monitoring in sports and healthcare applications.

## 1. Introduction

Gait and posture disorders are widespread and can significantly impact an individual’s quality of life, making effective monitoring crucial. Proper gait monitoring across different activities, such as standing, walking, jogging, and running, not only helps in identifying the underlying causes of these disorders but also plays a vital role in assessing the effectiveness of treatments and rehabilitation strategies. By closely tracking changes in gait patterns during these various activities, healthcare providers can detect early signs of neurological, musculoskeletal, or systemic conditions, allowing for timely intervention. Continuous monitoring provides valuable data for personalizing treatment plans, optimizing recovery, and preventing further complications. In rehabilitation, accurate gait analysis is essential for designing adaptive therapies that enhance mobility across different levels of physical activity. This comprehensive approach to gait monitoring is indispensable in clinical practice and research, contributing to better patient outcomes and a deeper understanding of various medical conditions. Besides injuries and monitoring, gait analysis is also important as it helps athletes refine their technique, prevent injuries, and improve overall performance.

A sensor attached to the knee can provide critical information about motion intention and gait patterns, offering valuable data for clinical diagnosis and assisting patients during walking. Among the physical sensing techniques, vision recognition and inertial measurement unit (IMU)-based sensors are widely used to interpret human motions [1,2,3,4]. However, these methods have notable drawbacks. Camera-based vision recognition raises privacy concerns and can be affected by varying lighting conditions and obstructions. IMU systems, while useful, are bulky, inflexible, and often a cause of discomfort. They also require extensive calibration and data processing, which can complicate their integration into wearable devices. Furthermore, traditional pressure sensors, while effective in measuring force, often lack the ability to provide detailed motion data and can be affected by environmental factors such as temperature and humidity. In contrast, a triboelectric flexible sensor attached to the knee, which directly converts motion states into electrical signals, offers significant advantages. This approach simplifies the sensory structure, providing a more straightforward, comfortable, and reliable solution for gait monitoring.

Hydrogels are favored for wearable sensors due to their flexibility, softness, and biocompatibility [5,6,7,8,9]. Unlike rigid sensors, hydrogel-based sensors offer greater comfort and adaptability, making them ideal for dynamic movements and long-term wear. They provide a conformal fit and reduce irritation, enhancing user comfort. Previous studies have used multiple rigid sensors, such as IMUs and pressure sensors, for accurate gait monitoring [10,11,12]. In contrast, hydrogel-based devices can achieve comparable performance with a single, flexible sensor, reducing complexity and enhancing wearability. However, it is important to recognize that while hydrogel-based sensors excel in flexibility and comfort, they are not without their limitations. For example, environmental factors such as temperature and humidity may affect their performance, potentially leading to signal drift or degradation. Additionally, the signal accuracy of hydrogel sensors, while promising, may not match the precision typically provided by IMU sensors, particularly in high-performance applications where fine-grained motion tracking is essential. These limitations highlight the need for continued optimization of hydrogel sensors to ensure their robustness and reliability in real-world conditions.

To incorporate desirable effects into flexible sensors, such as conductivity, environmental stability, biocompatibility, and mechanical strength, various organic and inorganic dopants are incorporated into hydrogels [13,14,15,16]. These dopants can be integrated through methods such as in situ polymerization, blending, or impregnation, depending on the specific requirements and applications of the final conductive hydrogel. MXenes, a class of two-dimensional transition metal carbides, nitrides, or carbonitrides, are being increasingly incorporated into hydrogels due to their unique properties. These include enhanced electrical conductivity, improved mechanical strength, increased thermal conductivity, superior electromagnetic interference (EMI) shielding, chemical stability, biocompatibility, versatility, and functionalization potential [17,18,19]. Additionally, MXenes impart photothermal and photocatalytic properties to hydrogels [20,21,22]. By incorporating MXenes, researchers can develop advanced materials that combine electrical, mechanical, and functional properties tailored for specific high-performance applications. To achieve the benefits facilitated by incorporating MXenes, this work focuses on the inclusion of MXenes into a hydrogel.

Our wearable device provides a leap forward in the field of gait monitoring. By incorporating an MXene-based hydrogel sensor in a very compact electronic device, we address the limitations associated with current devices. The sensor’s ability to generate electrical signals through mechanical strain allows for accurate and continuous monitoring of gait dynamics with minimal power consumption. This innovation eliminates the need for multiple sensors and complex wiring, resulting in a lightweight, comfortable device that integrates seamlessly into everyday wear. The small-size microcontroller enhances the device by providing efficient data processing and wireless transmission capabilities within a small, compact form factor. This integration simplifies the overall system of gait monitoring. Our device offers a more practical and user-friendly solution compared to current devices, making it well suited for continuous, real-time gait analysis for various applications, including healthcare and athletic performance monitoring.

Artificial intelligence (AI) has made substantial contributions to the evolution of wearable devices, transforming their functionalities and applications across diverse domains [23,24,25]. The traditional methods of analyzing sensory information typically encompass classical signal processing methods, statistical analysis approaches, pattern recognition algorithms, time series analysis, and even manual calibration and validation. These methods are often well established and can be effective for many applications, but they may not capture the complexity that contemporary deep learning techniques can manage. Deep learning involves training artificial neural networks to model complex patterns in data. Deep learning has demonstrated substantial potential in various fields such as image processing, natural language processing, speech recognition, human activity recognition, healthcare, and finance. Deep learning enhances the analysis of sensor signals by learning and extracting complex features from data. Therefore, deep learning offers a promising and viable solution to attain high analysis accuracy while maintaining low computational costs for wearable devices.

To date, there are several articles on deep learning-based wearable sensors for gait analysis using conventional inertial measurement units (IMUs) [26,27,28,29]. However, very few studies have explored deep learning approaches for hydrogel sensors. For example, Hang et al. developed a polyacrylamide hydrogel strain sensor with remarkable stretchability, high sensitivity, and excellent cyclic durability [30]. This wearable strain sensor can detect various human motions and transmit data to a smartphone via an integrated readout and a wireless module. Additionally, they fabricated a smart glove by incorporating multiple strain sensors and the corresponding circuitry. This smart glove can interpret and recognize American Sign Language and can wirelessly control a robotic hand through the hand gestures. However, the concept of machine learning is absent here. Moreover, the overall device suffers from poor aesthetics, discomfort, and bulkiness. He et al. developed a nanocomposite organohydrogel strain sensor to monitor human movements such as going upstairs, going downstairs, walking, and running, utilizing a two-level fully connected neural network (FCNN) [31]. They classified and recognized different motions using the FCNN deep learning model, achieving a classification accuracy of 87%. They directly connected their sensor to a Bluetooth module and transmitted the data to a phone. Overall, their device lacks portability, digital integration, high accuracy, and user-friendliness. On the other hand, there are several wearable devices incorporating AI and accelerometers for gait and health monitoring [32,33,34,35]. For instance, Matin et al. developed a real-time online assessment and mobility monitoring (ROAMM) system using smartwatches for remote health monitoring and mobility tracking [35]. They integrated a machine learning approach into the digital watch’s server software, allowing for real-time data retrieval and extensive analysis. However, the ROAMM framework using smartwatches offers promising advancements in remote health monitoring but faces key limitations. Battery life constraints may disrupt continuous data collection, while privacy concerns and security measures add complexity. Consumer-grade smartwatches may not offer the precision needed for high-level health assessments. For example, accelerometers may not detect subtle movements accurately, which is crucial for tasks such as fall detection. This limitation can compromise the quality and reliability of the data. This framework’s reliability could be affected by environmental factors like signal interference, GPS inaccuracy in dense urban areas, and connectivity issues in low-signal zones. These factors can impact the accuracy of location tracking and activity monitoring, undermining the quality of the data. Overall, compared to the studies mentioned above, our wearable device, which uses a polyacrylamide–lithium chloride–MXene (PLM) hydrogel sensor, offers a unique combination of flexibility, machine learning integration, wireless transmission, reliability, and compactness.

This paper presents a novel wearable device for gait monitoring through the development of a polyacrylamide–lithium chloride–MXene (PLM) hydrogel sensor integrated into a knee brace. The PLM hydrogel combines the advantages of flexibility, mechanical strength, and self-powering capabilities, achieved by incorporating MXenes into a polyacrylamide–lithium chloride matrix. The sensor’s ability to generate electrical signals from mechanical strain facilitates accurate and continuous gait monitoring with minimal weight. The system employs a compact microcontroller board for efficient data capture and analysis, with in-built wireless transmission. This integrated solution simplifies the traditional multi-sensor approach, enhancing user comfort and device practicality. A 1D convolutional neural network (CNN) is used for classifying different gait activities, demonstrating high accuracy and reliability. The wearable device offers a significant advancement in wearable technology and soft sensors, with applications in healthcare and sports monitoring.

## 2. Materials and Methods

### 2.1. Materials and Reagents

Acrylamide (AM), N,N,N′,N′-tetramethyl ethylenediamine (TEMED), N,N′-methylenebisacrylamide (MBA), ammonium persulfate (APS), and lithium chloride (LiCl) were obtained from Sigma-Aldrich (Melbourne, Australia). Sylgard 184 A-base and 184 B-curing reagent were purchased from Dow Chemical (Altona, VIC, Australia). Ti_3_C_2_T_x_ (MXene) multilayer nanoflake (thickness 100–200 nm, purity 54–68 wt%) was provided by Jiangsu XFNANO Materials Tech Co., Ltd. (Nanjing City, Jiangsu Province, China). Commercial polyurethane (PU) and polyethylene (PET) tapes were purchased from RS (Smithfield, NSW, Australia) A knee brace was purchased from the chemist warehouse (Melbourne, VIC, Australia) These materials were utilized without additional purification. Ultrapure water was employed in all experiments.

### 2.2. Preparation of the PAM-LiCl-MXene Hydrogel

We followed a similar procedure to synthesize the PAM-LiCl-MXene hydrogel to that documented in our prior research [1]. Initially, to attain a homogeneous dispersion of MXene in water, 100 mg of MXene nanosheet was ultrasonically dispersed in 10 mL of ultrapure water in an ice-water bath. The vial containing the MXene solution was kept in an inert environment and wrapped in aluminum foil to minimize light-induced oxidation. Subsequently, acrylamide powder (2.35 g), LiCl (3M), and 0.5 mL MXene nanosheet solution (10 mg mL^−1^) were added to 15 mL ultrapure water to create the AM-LiCl solution. Then, the mixture was vigorously stirred for 1 h at 80 °C under a nitrogen atmosphere. After that, MBA (0.00141 g) was added into the PLM homogeneous solution and stirred for 10 min. Then, the stirring and temperature were turned off to allow the solution to cool down to room temperature. Next, APS (0.004 g) was added to the solution and stirred for 1 min. Then, the homogeneous solution was poured into a self-designed dog-bone-shaped acrylic mold. The size of the hydrogel was determined following the ASTM Type 3 method. The resultant hydrogel had a thickness of 1 mm.

### 2.3. Preparation of the Triboelectric Layers

As a tribo-negative layer, PDMS film was prepared by fully mixing the Sylgard 184 A-base and 184 B-curing reagent at a weight ratio of 10:1, followed by spin coating on a glass plate at 1000 rpm for 1 min and curing at 80 °C for 2 h. The obtained PDMS film had a thickness of 500 μm. Then, the PDMS film was peeled off from the glass plate and cut to a size of 70 mm × 25 mm. Next, as a tribo-positive layer, commercial PU tape with a thickness of 0.21 mm was cut to the same size as the PDMS film.

### 2.4. Assembly of the PLM Sensor

The PAM hydrogel was sandwiched between two PDMS films to form the bottom-part PDMS/PAM layer of the sensor. To create an air gap, four PDMS spacers with a height of 2 mm were placed on the four corners of the top of the PDMS/PAM layer. Finally, PU film was attached to the spacer to form the upper layer of the TENG sensor. Here, the PDMS film, PAM hydrogel, and PU film are all highly flexible materials. The edges of the DH-TENG were sealed using commercial PET tape. The weight and dimensions of the PLM sensor are listed in the Supplementary Material (Table S2).

### 2.5. Wearable Device

#### 2.5.1. Circuit Diagram

**Figure 1:**
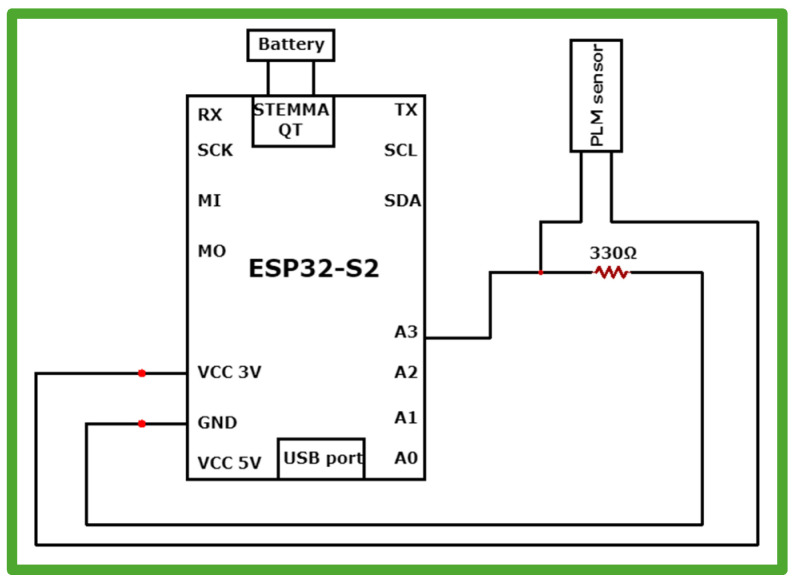
Schematic circuit diagram of the wearable device.

The schematic in Figure 1 illustrates the connection between the ESP32-S2 microcontroller and the prepared PLM sensor, designed to capture and process gait motion signals. The sensor’s output is routed through a 330-ohm resistor to Analog-to-Digital Converter (ADC) pin A3 on the ESP32-S2, enabling the microcontroller to digitize the analog signal generated by the sensor. This resistor likely serves to limit current or as part of a voltage divider circuit, ensuring the signal falls within a safe and measurable range for the ADC. This setup is integral to the systems that monitor and analyze signals from the PLM sensor, particularly in wearable or low-power applications.

#### 2.5.2. Device Assembly

**Figure 2:**
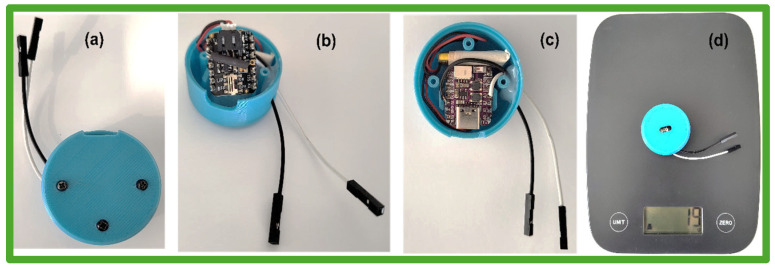
Photographs of the enclosure with the electronic circuit. (**a**) Enclosed. (**b**) and (**c**) Internal components. (**d**) Weight (19 g).

A three-dimensional printed, lightweight, and portable enclosure was created to enable the device to be carried by a human during gait monitoring. With a small diameter of 34 mm, this enclosure incorporates the circuit board and a polymer lithium-ion battery, offering a compact and lightweight configuration as shown in Figure 2.

The dimensions and weights of the individual components of the wearable device are listed in Tables S1 and S2 (see Supplementary Material).

#### 2.5.3. Microcontroller

The ESP32-S2 WiFi Dev Board (NSW, Australia) is a compact and powerful development board, ideal for wearable projects that require wireless connectivity. At its core, it uses a highly integrated, low-power, 2.4 GHz Wi-Fi System-on-Chip (SoC) microcontroller solution. The microcontroller offers 4 MB of Flash memory and 2 MB of PSRAM. The board features 13 GPIO pins, an 8-bit analog output DAC, and a 3.3 V regulator capable of a 600 mA peak output.

#### 2.5.4. Battery and Resistor Connection 

A standard 3.7 V 120 mAh lithium-ion battery was plugged into the JST plug on the end of a LiPoly charger. As a voltage divider, a 330 Ω resistor was soldered between analog pin A3 and the ground pin (GND) of the device.

#### 2.5.5. Assembly of the Smart Knee Brace

**Figure 3:**
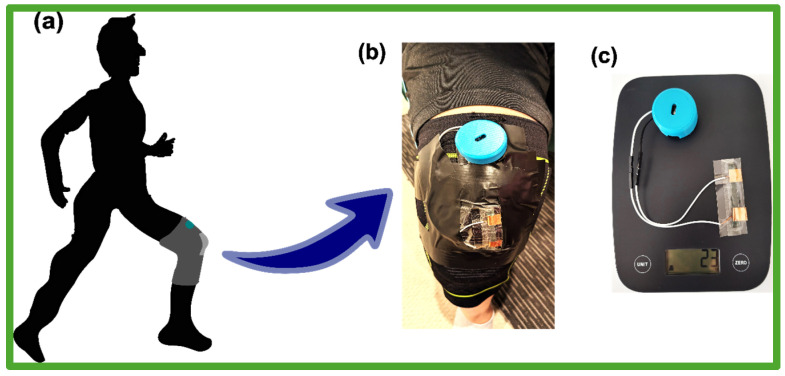
Knee brace. (**a**) Illustration of a human body wearing the knee brace. (**b**) Photograph of the knee brace with the wearable device. (**c**) Weight of the wearable device.

Figure 3 provides a comprehensive view of the knee brace designed for gait monitoring. Figure 3a illustrates the conceptual integration of the brace on a human body, specifically targeting the knee joint to facilitate real-time data collection during movement. Figure 3b offers a close-up photograph of the knee brace in its assembled state, with the wearable device comprising the electronic circuit-based enclosure and the PLM sensor strategically placed with the tapes to ensure both comfort and functionality. This secure attachment demonstrates the robustness of the setup, making it suitable for dynamic conditions such as walking or running.

Figure 3c shows the lightweight design of the system, where the wearable device weighs just 23 g. This minimal weight contributes to the overall comfort of the wearer, ensuring the brace can be used for extended periods without compromising the quality of gait analysis or causing user fatigue.

### 2.6. Experimental Measurement and Characterization

An Instron 30 KN Mechanical Tester (Instron, Norwood, MA, USA) controlled by a custom-built computer associated with a load cell of 50 N was used for all types of mechanical testing. The PLM sensor was loaded onto the tensile testing machine and uniaxially tested under a constant crosshead speed of 100 ${mm\;min}^{-1}$. All the experiments were carried out using stable hydrogel samples at 50% relative humidity and at approximately 20 °C room temperature. All the experimental results were calculated as the average of 3 or more tests.

The signal outputs of the PLM hydrogel-based sensor were measured by electrometer Model 6514, Keithley (Tektronix, Balwyn, VIC, Australia), using the four-probe method and a high-impedance probe of 100 MΩ. Analog voltage signals generated by the sensor on the knee brace were collected and processed by the wearable device. The signals were transmitted wirelessly to a computer with the help of an in-built Wi-Fi module. The 1D CNN models were developed in Python (3.10.12) with Tensorflow (2.17.1) and Keras (3.5.0). 

## 3. Results and Discussion

### 3.1. Preparation and Working Mechanism

**Figure 4:**
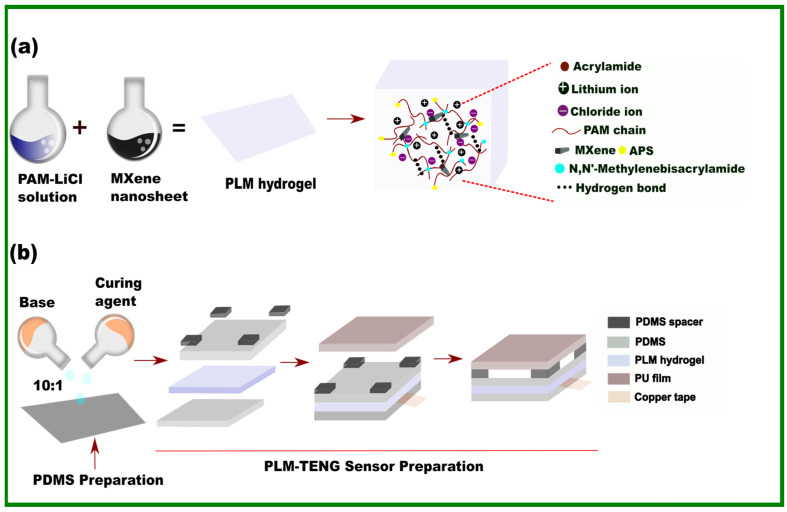
Preparation of the PLM sensor. (**a**) Preparation of the PLM hydrogel (left); internal morphology of the hydrogel (right). (**b**) PDMS preparation (left); assembly of the PLM sensor (right).

Figure 4 illustrates the step-by-step procedure for preparing the PLM hydrogel and assembling the PLM sensor. The process begins with the mixing of acrylamide and lithium chloride, followed by the incorporation of MXene into the PAM-LiCl solution to form a PLM hydrogel, as shown in Figure 4a (left). The inclusion of the PAM provides structural integrity and flexibility due to its robust polymeric network. Lithium chloride enhances the hydrogel by imparting high ionic conductivity, large elongation, antifreezing properties, improved moisture retention capacity, and superior electrochemical performance. MXene, a two-dimensional material, contributes high electrical conductivity and mechanical strength. The entire conductive network is developed through chemical cross-linking, physical cross-linking, and supramolecular interactions. Figure 4a (right) depicts the internal morphology and bonding within the PLM hydrogel network. Figure 4b shows the assembly process in sequential steps, from left to right: the preparation of the PDMS layers by mixing the base and curing agent in a 10:1 ratio, layering the prepared hydrogel between PDMS (polydimethylsiloxane) layers with spacers on top, adding PU (polyurethane) films, and using copper tape for electrical connections. This composite structure leverages the unique properties of each component to create a flexible, conductive material suitable for advanced electronic devices. The integration of these materials results in a highly versatile and innovative solution, ideal for applications in wearable electronics, sensors, and soft robotics, showcasing both durability and high performance.

**Figure 5:**
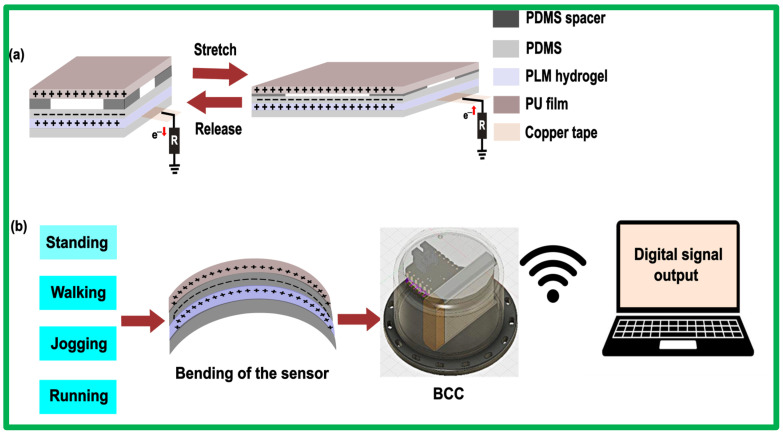
Working mechanism of the PLM sensor-based gait monitoring system. (**a**) Working principle of the PLM sensor during a stretch–release cycle. (**b**) System-level block diagram of the gait monitoring system, showing analog signals from the four activities (blue), processing and wireless transmission (green), the digital signal output, and the machine learning algorithm run by the computer (yellow).

Figure 5 shows the working principle of the PLM sensor. The core of this sensor consists of a conductive PLM hydrogel as the primary electrode, encapsulated within tribo-negative PDMS layers, with a PU film serving as the top-most tribo-positive layer as shown in Figure 5a. The integration of MXene into the polyacrylamide–lithium hydrogel ensures that significant electrical conductivity is maintained even under extreme elongation, ensuring exceptional durability of the sensing units. Different levels of axial tensile strain in both the PDMS and conductive hydrogel layers cause continuous variation in the contact area between the two during stretch–release cycles. During stretching, the layers come into contact, causing the PDMS and PU surfaces to become electrically charged due to contact electrification. According to the triboelectric series, PDMS has a strong affinity for negative charges, whereas PU tends to lose negative charges. Thus, surface charge transfer occurs between the PDMS and PU layers due to the triboelectric effect. When the PDMS and PU layers separate during release, a potential difference is generated between the hydrogel layer and the ground. Simultaneously, due to electrostatic induction, the negative charges in PDMS induce positive charges in the hydrogel layer. This combined effect of potential difference and electrostatic induction causes free electrons to move from the PLM hydrogel layer to the ground, resulting in an instantaneous current. Figure 5b provides an overview of the process flow of both hardware and software, starting with four activities of standing, walking, jogging, and running, then analog signal acquisition from bending of the sensor, followed by conditioning and processing through the wearable device, and ending with the wireless transmission to the computer. The computer is embedded with a machine learning algorithm for digital monitoring of gait patterns. This microcontroller with both computational and serial communication capabilities calibrates the conditioned signals for an onboard wireless transceiver, which transmits the signals to a computer. The computer, integrated with a 1D CNN machine learning algorithm, classifies time-series data into four distinct gait patterns.

### 3.2. Mechanical and Electrical Characterization

**Figure 6:**
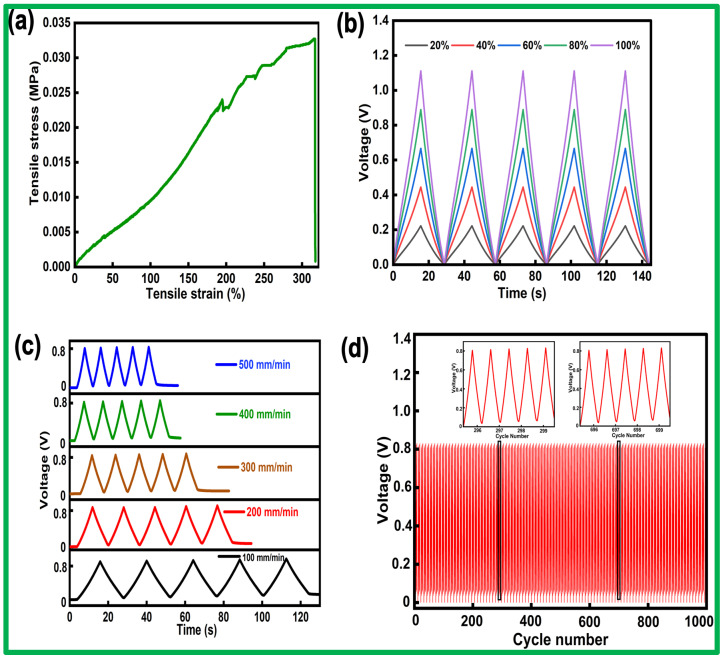
Mechanical and electrical performance of the PLM sensor. (**a**) Tensile stress–strain characteristics. (**b**) Generated voltage signals with different tensile strains (20%, 40%, 60%, 80%, and 100%). (**c**) Generated voltage signals at different stretching rates (from 100 to 500 mm min^−1^) at a fixed strain of 80%. (**d**) Mechanical durability test for up to 1000 continuous stretch–release cycles at 80% strain.

The mechanical performance of the PLM sensor was evaluated using a computer-controlled universal material testing machine operating at a constant speed of 100 mm ${min}^{-1}$. Figure 6a illustrates the tensile properties of the PLM sensor, revealing a maximum strain of 317% and a peak tensile stress of 0.0327 MPa. A photograph of the tensile testing of the PLM TENG sensor is shown in Supplementary Material (Figure S1).

Next, we investigated the voltage output of the PLM sensor under varying axial strains. We employed an integrated system comprising a computer-controlled universal testing machine and a four-probe Keithley multimeter to precisely measure its voltage responses at different strains. Initially, the two terminals of the dog-bone-shaped PLM TENG sensor were individually secured between the clips of the fixtures in the universal testing machine. The tensile fixtures incorporated two pairs of parallel clips, consisting of a stainless-steel grip combined with an external rubber pad. This insulating rubber pad prevents resistance leakage from the attached sensor. Subsequently, a pair of soldered copper wires (four in total) from each edge of the PLM TENG sensor were connected to the four probes of the multimeter, utilizing the four-wire resistance method. This combined setup of the material testing and voltage measurement device allowed us to precisely control the tensile strains and concurrently obtain the corresponding generated voltage values.

The generated voltage profiles under a stretch speed of 100 mm ${min}^{-1}$ were recorded at various strains of 20%, 40%, 60%, 80%, and 100% (Figure 6b). The output signals exhibit remarkable stability and uniformity across various strains and demonstrate a proportional relationship between the tensile strain and signal amplitude. This behavior underscores the advantages of the structural design and the reliable electrical output of the sensing unit. In addition, the dependence of the voltage amplitude under a constant strain of 80% for stretching speeds ranging from 100 to 500 mm ${min}^{-1}$ is shown in Figure 6c. The voltage exhibits rapid changes over five cycles at varying stretching speeds, devoid of any noticeable hysteresis or fluctuation. This demonstrates the swift responsiveness of the electrical behavior of the PLM sensor to the applied strain.

The mechanical durability of the PLM sensor is a significant characteristic for practical applications requiring prolonged use. As illustrated in Figure 6d, the sensing unit exhibited negligible changes in output voltage over 1000 continuous stretch–release cycles at 80% strain. Notably, 80% strain far exceeds the requirements for most gait monitoring applications.

### 3.3. Development of the Deep Learning Model

Deep learning models, particularly convolutional neural networks (CNNs), play a crucial role in gait monitoring analysis by providing high accuracy and flexibility in detecting movement patterns. Unlike traditional approaches that require manual feature selection, deep learning automatically learns detailed features directly from the raw sensor data, leading to better recognition of gait variations and activity classification. These models are highly effective in handling differences in walking styles and environmental changes, making them well suited for healthcare, rehabilitation, and sports applications. Integrating deep learning into gait analysis enhances the ability to monitor and assess movement more precisely, supporting personalized healthcare and sports monitoring.

**Figure 7:**
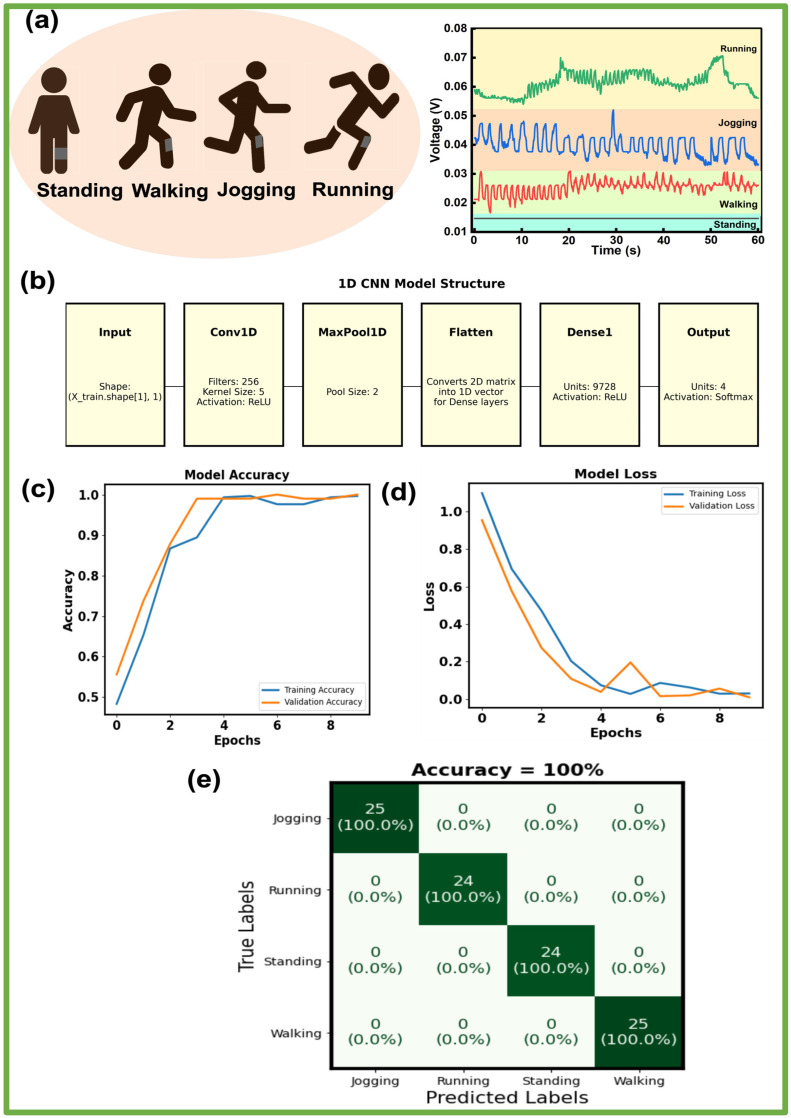
Gait identification using the 1D CNN model. (**a**) Voltage acquisition from four gait patterns: standing, walking, jogging, and running. (**b**) Model structure. (**c**) Model accuracy. (**d**) Model loss. (**e**) Confusion map of the accuracy prediction for the four activities.

In this work, the raw data from the knee brace with the PLM hydrogel sensor, which contained information about the gait pattern and output voltage, were fed into the training 1D CNN model directly without any additional data processing steps. Figure 7a shows the gait pattern identification of a single participant wearing a single sensor attached to a knee brace with the 1D CNN method. A dataset was built from the participant performing four activities, standing, walking, jogging, and running, across a 100 m corridor within ~60 s. The whole dataset was divided into the training set (60%), testing set (20%), and validation set (20%).

Figure 7b shows the overall 1D CNN model structure of this work. Considering the trade-off between the number of samples and window length, the highest accuracy was achieved with frames consisting of 80 samples, which corresponds to 4 s of data sampled at 20 Hz. With a hop size of 40 samples, the frames overlap by 40 samples (or 2 s). Using these parameters, the data were fed into a 1D CNN where each input segment contained 80 samples, and each subsequent segment began 40 samples after the previous one, allowing the frames to overlap and capture additional contextual information. The 1D convolutional neural network (CNN) architecture was implemented using TensorFlow/Keras to classify the time-series data into four distinct categories. The network started with a convolutional layer featuring 256 filters with a kernel size of five and utilized the ReLU activation function to identify spatial patterns within the sequences. This was followed by a max pooling layer with a pool size of two, which reduced feature map dimensions while preserving key features. The pooling layer’s output was flattened into a 1D vector for processing by the dense layers. The model included a dense layer with 9728 units and ReLU activation for complex feature learning. The final dense layer, with four units and a softmax activation function, classified the data into one of four classes, generating probability distributions for each class. This architecture effectively integrates feature extraction, dimensionality reduction, and classification for sequence-based tasks.

The model was compiled with the Adam optimizer at a learning rate of 0.001, optimizing learning rates during training for improved convergence. Sparse categorical cross-entropy was chosen as the loss function, which is suitable for multi-class classifications. Accuracy was used as the primary metric to gauge performance, representing the proportion of correct classifications. Training was conducted over 10 epochs with validation on a separate set to monitor performance on unseen data and assess the generalization.

Figure 7c illustrates the training and validation accuracy of our developed 1D CNN model. The X axis and Y axis represent the number of epochs and accuracy range, respectively. The blue line shows a sharp increase in accuracy in the initial epochs, reaching close to 100% accuracy by around epoch 5 and remaining stable afterward. Similarly, the validation accuracy rises quickly and aligns closely with the training accuracy, reaching 100% by the final epochs. The close alignment between the training and validation accuracy curves suggests that the model generalizes well without overfitting. Figure 7d presents the training and validation loss for our 1D CNN model over 10 epochs. The training loss shown by the blue line starts at a high value, around 0.95, and consistently decreases as the epochs progress, reaching down to 0.02 by the ninth epoch. The validation loss, represented by the orange line, also begins with a relatively high value but drops more rapidly than the training loss within the first few epochs. However, there is a slight fluctuation around epoch 5, where the validation loss increases briefly before stabilizing. The consistent reduction in both training and validation loss suggests that the model is effectively learning without signs of overfitting or underfitting, as both metrics converge to a low value of 0.02. The overall accuracy of 100%, as shown by the confusion matrix in Figure 7e, reflects the model’s effective classification performance. Specifically, the model achieved 100% accuracy for jogging, running, standing, and walking activities. Overall, these graphs indicate a well-trained model with high accuracy and minimal loss, performing exceptionally well on both the training and validation datasets. The confusion matrix confirms this with a perfect classification outcome across all classes. This model demonstrates robust performance in classifying the activities accurately. In summary, this proposed smart knee brace offers a range of functionalities suitable for energy harvesting and for digital gait sensing recognizing four activities. Future investigations could provide a more detailed understanding of user interactions across various domains, including real-time identity recognition, smart home applications, sports monitoring, and healthcare monitoring.

## 4. Conclusions

In this study, we developed and characterized an advanced smart knee brace incorporating a polyacrylamide–lithium chloride–MXene (PAM-LiCl-MXene)-based hydrogel. The integration of this sensor with a lightweight ESP32-S2 microcontroller system enabled real-time gait monitoring with exceptional accuracy.

The mechanical and electrical properties of the PLM sensor demonstrated impressive stretchability, with a maximum strain of 317% and stable voltage output under varying tensile strains. The sensor’s mechanical durability, shown through 1000 stretch–release cycles with minimal performance degradation, highlights its suitability for continuous use in wearable applications. The high electrical conductivity and flexibility of the hydrogel, enhanced by MXene, ensure robust performance even under dynamic conditions.

A 1D convolutional neural network (CNN) was employed to classify gait patterns, achieving a high accuracy of 100%. This performance underscores the effectiveness of combining triboelectric sensing with deep learning for gait recognition. The system’s lightweight design, with the entire setup weighing just 23 g, ensures user comfort and practicality.

The use of a smart knee brace to house the lightweight wearable device and the PLM sensor demonstrates the feasibility of integrating advanced sensing technologies into wearable and portable formats. The knee brace design ensures that the sensor is positioned optimally to capture gait patterns accurately while remaining unobtrusive to the user’s movement. This integration also facilitates the real-time monitoring of gait patterns, providing valuable data for healthcare professionals and researchers.

Future research will focus on further refining the material properties and structural design to enhance the sensor’s performance and longevity. Additionally, efforts will be directed towards miniaturization and the integration of additional functionalities, such as multi-modal sensing capabilities to detect a wider range of motions and health parameters. Advanced machine learning algorithms will be explored to improve the data analysis accuracy and provide more comprehensive insights.

## Data Availability

Data are contained within the article.

## Supplementary Materials

The following supporting information can be downloaded at: https://www.mdpi.com/article/10.3390/s24227370/s1, Table S1: Listing and technical specification of the individual components housed in the blue circular case; Table S2: Technical specification of the PLM-TENG sensor; Figure S1. Mechanical testing of the PLM-TENG sensor.
