# Supplementary material for: AI-Aided Gait Analysis with a Wearable Device Featuring a Hydrogel Sensor
Source: Sensors (Basel). 2024 Nov 19;24(22):7370. doi: 10.3390/s24227370 (PMC11598565; doi:10.3390/s24227370)
Supplement: Supplementary file 1 [file sensors-24-07370-s001.zip › sensors-3296891-supplementary.pdf]

Supporting Information

A Machine Learning Approach for Gait Analysis by Using Polyacrylamide-Lithium Chloride-MXene Based Hydrogel

Saima Hasan[a], Brent Garry D'auria[a], M. A. Parvez Mahmud[b], Scott D. Adams[a], John Long[a], Lingxue Kong[c], Abbas Z. Kouzani[a]*

[a]*School of Engineering, Deakin University, Geelong, VIC 3216, Australia*
[b]*Faculty of Science, University of Technology Sydney, Ultimo, NSW 2007, Australia*
[c]*Institute for Frontier Materials, Deakin University, Geelong, VIC 3216, Australia*

AUTHOR INFORMATION
Corresponding Author
*E-mail: kouzani@deakin.edu.au

- **Listing of the individual components of the smart gait monitoring system:**

Table S1: Listing and technical specification of the individual components housed in the blue circular case.

Serial no.	Name of the components	Rating	Weight (g)	Dimension	Quantity
1	Polymer Lithium Ion Battery	3.7V, 120mAh	11	28mmx13mmx5mm	1
2	Adafruit QT Py ESP32-S2 WiFi Dev Board with STEMMA QT	N/A	2.1	21.8mm x 17.9mm x 5.7mm	1
3	LiPoly Charger	N/A	1.6	20.9mm x 17.9mm x 7.2mm	1
4	Jumper wire	N/A	N/A	N/A	4
5	Electronic resistor	330 Ohm	N/A	N/A	1
6	3D printed blue circular case	N/A	4	34mm x 18mm	1

Table S2: Technical specification of the PLM-TENG sensor.

Serial no.	Name of the components	Weight (g)	Dimension	Quantity
1	PLM-TENG sensor	4	70mm x 25mm x 3mm	1

➢ **Tensile testing of the PLM-TENG sensor:**

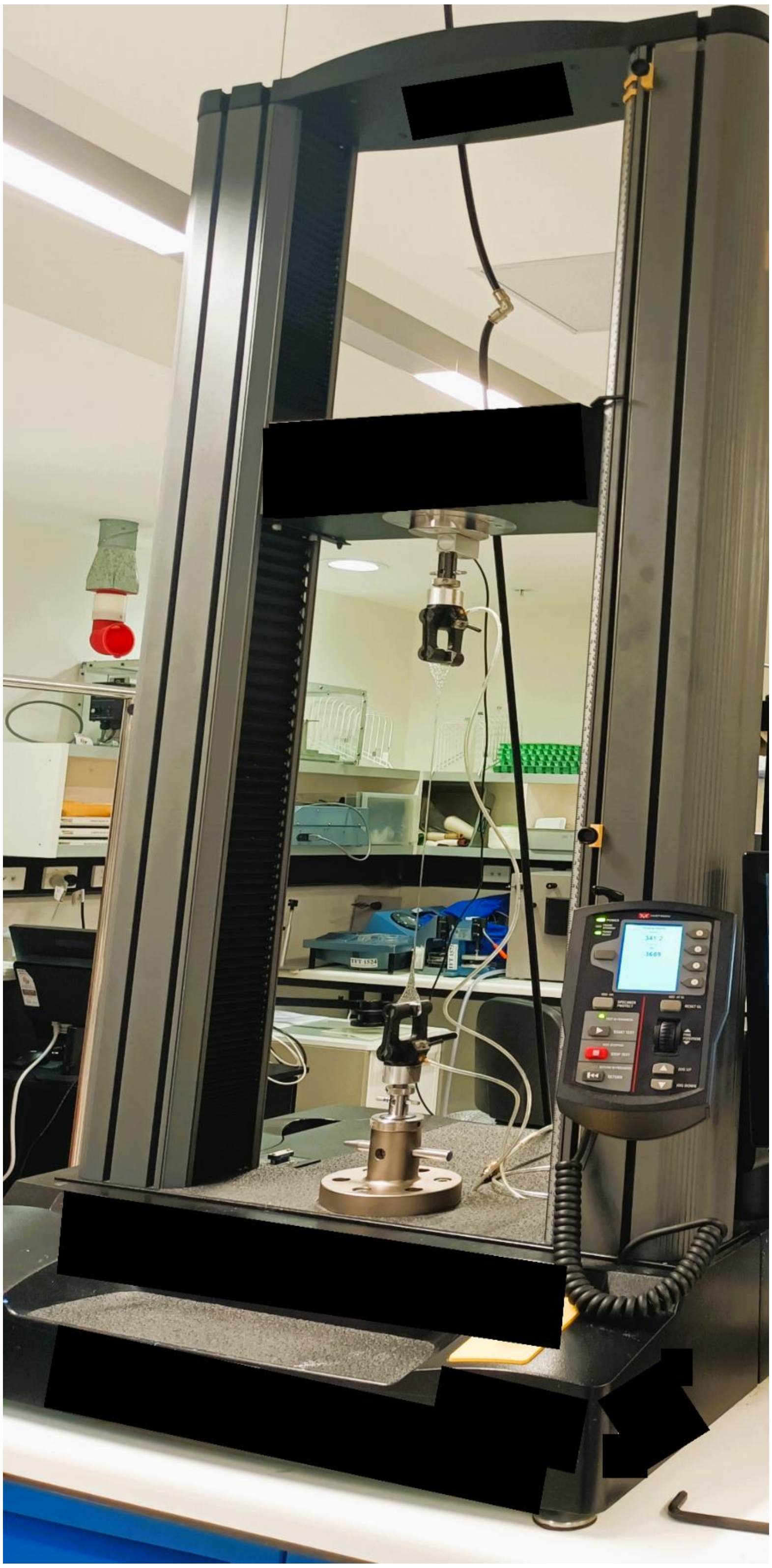

Figure S1. Mechanical testing of the PLM-TENG sensor. (For the privacy labels are hidden).
